# The Toxic Effects of Polychemotherapy onto the Liver Are Accelerated by the Upregulated MDR of Lymphosarcoma

**DOI:** 10.5402/2012/721612

**Published:** 2012-11-29

**Authors:** Alexandra V. Sen'kova, Nadezhda L. Mironova, Olga A. Patutina, Tatyana A. Ageeva, Marina A. Zenkova

**Affiliations:** ^1^Institute of Chemical Biology and Fundamental Medicine, Siberian Branch of Russian Academy of Science, Lavrentiev Avenue 8, Novosibirsk 630090, Russia; ^2^Novosibirsk State Medical University, Krasnyi Prospect 52, Novosibirsk 630091, Russia

## Abstract

Antitumor therapy of hematological malignancies is impeded due to the high toxicity of polychemotherapy toward liver and increasing multiple drug resistance (MDR) of tumor cells under the pressure of polychemotherapy. These two problems can augment each other and significantly reduce the efficiency of antineoplastic therapy. We studied the combined effect of polychemotherapy and upregulated MDR of lymphosarcoma RLS_40_ onto the liver of experimental mice using two treatment schemes. Scheme 1 is artificial: the tumor was subjected to four courses of polychemotherapy while the liver of the tumor-bearing mice was exposed to only one. This was achieved by threefold tumor retransplantation taken from animals subjected to chemotherapy into intact animals. Scheme 2 displays “real-life” status of patients with MDR malignancies: both the tumor and the liver of tumor-bearing mice were subjected to three sequential courses of polychemotherapy. Our data show that the strengthening of MDR phenotype of RLS_40_ under polychemotherapy and toxic pressure of polychemotherapy itself has a synergistic damaging effect on the liver that is expressed in the accumulation of destructive changes in the liver tissue, the reduction of the regeneration capacity of the liver, and increasing of Pgp expression on the surface of hepatocytes.

## 1. Introduction

Despite the significant progress in the therapy of hematological malignancies there are several problems that impede the use of the polychemotherapy for treatment of this group of diseases. The first one is the high intrinsic toxicity of multiple chemotherapies [1, 2] that may lead to the multiple-organ failure in particular liver dysfunction. The next is the strengthening of multidrug resistance (MDR) of tumor cells by cytostatic agents that are often characterized by the MDR phenotype even at baseline [3–5]. These two problems can augment each other and can significantly reduce the efficiency of antineoplastic therapy.

The resistance of tumor cells to chemotherapy can be a result of various processes: from active ATP-dependent transport of cytotoxic agents out of the cells executed by P-glycoprotein, the member of subfamily B of ABC-transporters [6, 7], to disorders in the apoptosis, mutations or downregulation of *p53* gene, impairing its proapoptotic function, and/or overexpression of *bcl-2* gene causing insensitivity of cells to proapoptotic stimuli [8–10].

Studies of pharmacokinetics of antitumor drugs suggest that they undergo biotransformation in the liver with the formation of toxic metabolites, which may cause hepatocyte damage following hepatic dysfunction [11, 12]. Hepatic dysfunction is one of the main factors limiting full adherence to the chemotherapy regimen by patients, because the development of complications in this organ due to chemotherapy can lead to lethal outcomes during cytostatic treatment. Although the researcher pays much attention to MDR phenotype acquiring by the tumor in the course of the treatment and separately to the influence of polychemotherapy onto the liver, little is known about the cumulative hepatotoxic effects evoked by cytostatics and by the tumor with MDR phenotype enhanced after exposure to multiple courses of chemotherapy.

Herein, we studied the combined toxic action of polychemotherapy by CHOP protocol, three components (cyclophosphamide, doxorubicin, and vincristine) of which are the substrates for ABC-transporters and the impact of upregulated MDR of lymphosarcoma RLS_40_ on the liver of experimental animals using two treatment schemes. In the course of the treatment under Scheme 1 the liver of tumor-bearing animals was subjected to the only one course of CHOP while tumor was consequently subjected to four courses of treatment. It was achieved by the retransplantation of tumor cells from the animals received one course of CHOP, to the healthy animals with intact livers. Then the animals were subjected to the next course of CHOP while the tumor cells at the same time were exposed to the second course. As a result four courses of polychemotherapy for the tumor were done but the liver has been exposed every time to the only one course. This experimental model is artificial, because it has no analogue in patients in clinical practice. We have developed this treatment scheme to evaluate the strengthening of the MDR of tumor and its contribution to the liver toxicity. In the course of the treatment under Scheme 2 tumor-bearing mice were exposed to three subsequent courses of CHOP. This experimental model represents the real picture in clinical practice in cancer patients undergoing multiple courses of chemotherapy.

Our data show that the toxic effect of polychemotherapy onto the liver is accelerated by the upregulated MDR of lymphosarcoma RLS_40_, and altogether they cause the increase in the destructive changes of the hepatocytes and the overall reduction in the regeneration capacity of the liver.

## 2. Methods

### 2.1. Cell Line

RLS_40_ cell line was obtained from RLS by selection of RLS tumor cells *in vitro* in medium supplemented with vinblastine at concentrations ranged from 5 to 40 nM and displayed high resistance to a wide range of cytostatics [13]. RLS_40_ cells were maintained in the Iscove's Modified Dulbecco's Medium (IMDM) supplemented with 10% fetal bovine serum (FBS) and 1% antibiotic antimycotic solution (10.000 *μ*g/mL streptomycin, 10.000 IU/mL penicillin, and 25 *μ*g/mL amphotericin; ICN, Germany) in the presence of 40 nM vinblastine in a humidified atmosphere containing 5% CO_2_ at 37°C and were regularly passaged to maintain the exponential growth.

### 2.2. Tumor Generation

RLS_40_ in ascitic form is permanent and maintained in CBA mice by regular passaging of tumor cells. In order to generate ascites tumors from primary cell culture 2 × 10^6^ cells/mL in 0.2 mL of the saline buffer were intraperitoneally injected into the abdominal cavities of CBA mice. For generating solid tumors from ascites intramuscular injections of 5 × 10^6^ cells/mL in 0.1 mL of solution were performed into the right thigh muscle of CBA mice.

### 2.3. Experimental Animals

The study was carried out on 10–14-week-old male CBA/LacSto (hereinafter, CBA) mice bred at the Institute of Cytology and Genetics (Siberian Branch, Russian Academy of Sciences, Novosibirsk, Russia). Mice were housed in groups of 8–10 individuals in plastic cages. The mice had free access to food and water and the daylight conditions were normal. Mice were sacrificed by cervical dislocation under ether narcosis. All animal procedures were carried out in accordance with approved protocol and recommendations for proper use and care of laboratory animals (European Communities Council Directive 86/609/CEE).

### 2.4. Tumor Transplantation and Design of Animal Experiments

#### 2.4.1. Experimental Scheme 1

Solid tumors RLS_40_ were induced via intramuscular injection of ascitic RLS_40_ cells (5 × 10^6^ cell/mL, 0.1 mL per mouse) suspended in the saline buffer into the right thighs of mice.

On day 7 after tumor transplantation each mouse bearing RLS_40_ was assigned to one of the two groups (*n* = 15 per group): the first group, control, received the saline buffer intraperitoneally; the second group received standard combination of cytostatics (CHOP protocol). The cytostatics were dissolved in the saline buffer directly before use and injected into the caudal vein in doses corresponding to 1/5 of LD_50_: cyclophosphamide (50 mg/kg), doxorubicin (4 mg/kg), and vincristine (0.1 mg/kg) (Lens-Farm). Prednisolone (5 mg/kg) (Nycomed) was intraperitoneally injected daily for 5 days. On day 7 after CHOP (day 14 after tumor transplantation) at the 1st passage and on day 10 after CHOP (day 17 after tumor transplantation) at the subsequent passages, the mice were sacrificed by cervical dislocation under ether narcosis and tumor tissues were used for the preparation of cell suspension. A portion of this suspension was used for the retransplantation (passages) into intact animals (*n* = 30) (5 × 10^6^ cell/mL; 0.1 mL per mouse); another portion was used for the preparation of primary cell culture and evaluation of gene expression profile. On day 7 after retransplantation of the tumor cells a portion of mice were subjected to the 2nd course of CHOP (*n* = 15); the control animals were left without treatment (*n* = 15). In total, four passages of tumors were carried out in mice followed by polychemotherapy.

Material for gene expression analysis was collected on day 7 after CHOP at the first passage of the tumor and on day 10 after CHOP during the other passages of the tumor. Material for histological analysis was collected on days 1, 3, and 7 after the treatment at the first passage of the tumor, on days 7 and 10 at the second and the third passages, and on days 7, 10, and 14 at the fourth passage of the tumor.

#### 2.4.2. Experimental Scheme 2

10–14-week-old male CBA mice were assigned into 3 groups (*n* = 45 per group). For generating of solid tumors RLS_40_ cells (10^6^ cells/mL, 0.1 mL per mouse) were transplanted intramuscularly into the right thighs of the animals of groups 1 and 2. Group 3 was left without tumor transplantation. On day 7 after tumor transplantation group 2 (tumor-bearing mice) and group 3 (mice without tumor) received a standard combination of cytostatics (CHOP protocol, see above). Group 1 (RLS_40_-bearing mice) was left without treatment. On day 7 after CHOP (day 14 after tumor transplantation), a portion of the mice were sacrificed by cervical dislocation under ether narcosis and tumor tissues were used for the preparation of primary cell culture. The remaining mice both with the transplanted tumor and without the tumor received 2nd and 3rd courses of CHOP with an interval of 7 days. On day 7 after 2nd and 3rd courses of CHOP (days 21 and 28 of tumor development) mice were sacrificed.

Material for histological study was collected on days 1, 3, and 7 after each course of chemotherapy. Material for gene expression analysis was collected on day 7 after each course of chemotherapy.

The tumor size was determined every other day using caliper measurements in three perpendicular dimensions. Tumor volumes were calculated as *V* = (*π*/6 × length × width × height). The inhibition of tumor growth was estimated as follows: [(mean tumor weight_control_ − mean tumor weight_experiment_)/mean tumor weight_control_] × 100%.

### 2.5. Primary Cell Culture of RLS_40_ Tumor

Isolation of primary cell culture from RLS_40_ solid tumor was carried out by separation of cell suspension obtained after homogenization of solid tumors through Lymphocyte Separation Medium (LSM). The cells were cultured in IMDM supplemented with 10% fetal bovine serum (FBS), 1% antibiotic antimycotic solution (10.000 *μ*g/mL streptomycin, 10.000 I.U./mL penicillin, and amphotericin 25 *μ*g/mL; ICN, Germany) at 37°C and 5% CO_2_ for 1 week until analysis.

### 2.6. RNA Isolation

Total RNA was isolated from cells according to the published protocol [14]. RNA concentration in the samples was measured by absorbance at 260 and 280 nm using a Bio-Mate 3 (Thermo Electron Corporation) spectrophotometer.

### 2.7. RT-PCR Analysis

The primers and RT-PCR conditions were described earlier [13, 15]. *β-Actin*-specific product was used as an internal control. *Mdr1a-* and *mdr1b*-specific products were amplified for 26 and 24 cycles. *β-Actin*-specific primers were added to the reaction mixture at the third and the first cycles, respectively. *Bcl-2-* and *p53*-specific products were amplified for 31 cycles; *β-actin*-specific primers were added to the reaction at the sixth cycle. Amplification was performed as follows: initial step 95°C for 5 min, then 24, 26 or 31 cycles of 94°C for 1 min, 57°C for 1 min, and 72°C for 1 min. PCR products were analyzed by electrophoresis in 8% polyacrylamide gel using TBE×1 as running buffer, visualized by ethidium bromide staining, photographed in UV light, and densitometrically quantified using Gel-Pro Analyzer 4.0. The data were presented as a ratio of specific gene expression level to *β-actin* expression level.

### 2.8. Measurement of Cytokine Levels in the Blood Serum

The levels of TNF-*α*, IL-1*α*, IL-6, IFN-*α*, and IFN-*γ* in the blood serum of mice bearing RLS_40_ without any treatment and treated with two courses of CHOP were measured using Mouse TNF-alpha, IL-1-alpha, IL-6, and IFN-gamma Colorimetric ELISA Kits (ThermoScientific, USA) according to the manufacture's protocols. The level of IFN-*α* was measured using Mouse Interferon Alpha (Mu-IFN-*α*) ELISA Kit (ThermoScientific, USA).

### 2.9. Histological Study

For morphological and morphometric analysis, tumor and liver samples of experimental animals were fixed in 10% neutral-buffered formaldehyde, routinely processed and embedded in paraffin [16, 17]. Paraffin sections (up to 5 *μ*m) were stained with hematoxylin and eosin, microscopically examined and scanned.

Stereological quantification was performed by point counting, using closed test system at a magnification ×400. Used test system has 16 straight-line segments and 25 testing points in a testing area equal to 1.16 × 10^5^ *μ*m^2^ and was applied for microscopic examination and morphometric measurements [18–20]. Ten or fifteen random fields were studied in each tissue specimen.

Morphometric analysis of the liver was performed and the volume densities (Vv) of normal liver parenchyma, hepatic lymphoma infiltration, hepatocytes with degenerate, and necrotic changes (destructive changes = degeneration + necrosis) were evaluated. The volume density (Vv) representing the volume fraction of tissue occupied by each compartment was determined from the points lying over these structures and calculated using the following formula: Vv = (*P*_structure_/*P*_test_) × 100%, where *P*_structure_ denotes the number of points over the structure and *P*_test_ represents the total number of test points, 25 in this case.

Numerical density of binuclear hepatocytes (Nv) indicating the number of cells in the unit of tissue volume was determined by counting the number of binuclear cells within the test area (grid with a square of 1.16 × 10^5^ *μ*m^2^).

Immunohistochemical staining of P-glycoprotein (Pgp) (ab 3364, Abcam, England) in tumor and liver sections was carried out according to the manufacture's protocol [21, 22]. Index of Pgp staining used for assessment of staining intensity of tumor sections was calculated as follows: In(Pgp) = (number of Pgp+ cells/total cell number) × 100%, where the total cell number was at least 100–150 cells for each tumor sample [23, 24]. The intensity of Pgp staining of the liver sections was scored on a scale of 0–3+, in which 0 was no staining, 1+ weak positive staining, 2+ moderate positive staining, and 3+ strong positive staining [25].

### 2.10. Statistics

The data were statistically processed using the Student's *t*-test (two-tailed, unpaired); *P* < 0.05 was considered statistically significant.

## 3. Results

### 3.1. Tumor Model

We used a tumor model with a proved predisposition to the strengthening of MDR status after cytostatic exposure-murine lymphosarcoma RLS_40_ which is related to human diffuse large B-cell lymphoma. RLS_40_ was obtained by selection of RLS tumor cells *in vitro* in medium supplemented with vinblastine at concentrations ranged from 5 to 40 nM and exhibits high resistance to a wide range of cytostatics [13]. Parental RLS tumor cell line is characterized by an increased expression of *mdr1b *and *bcl-2* genes and decreased level of *p53* gene [13]. RLS_40_ tumor is resistant to apoptosis induction and is characterized by the upregulated *mdr1a/1b* genes' expression [15]. The MDR of RLS_40_ corresponds to the tumor status in patients after chemotherapy or to the status of a tumor initially resistant to standard chemotherapy protocols used as the first line therapy in the majority of hematological malignancies.

### 3.2. Design of the Experiment

**Figure 1:**
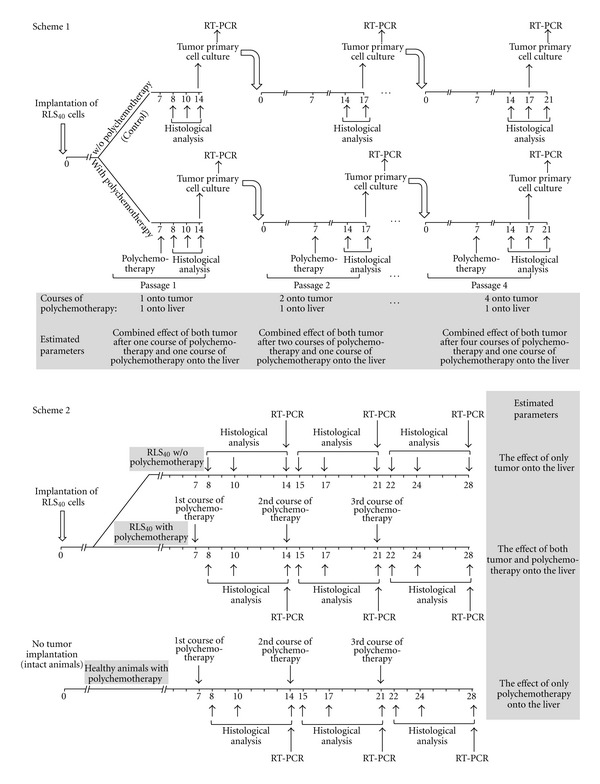
Experimental schemes of the treatment of mice bearing RLS_40_. Scheme 1: tumor-bearing mice were exposed to polychemotherapy (passage 1) followed by preparation of primary tumor cell culture, implantation of these cells into healthy animals, and repeated polychemotherapy (passages 2, 3, and 4). Scheme 2: tumor-bearing mice was exposed to three subsequent courses of polychemotherapy.

The aim of our study was to investigate the effect of polychemotherapy (single and multiple) onto aggravation of the liver toxicity and the impact of the enhanced MDR of tumor into observed hepatotoxicity. For these purposes two experimental schemes were used (Figure 1). In the treatment using Scheme 1 mice with intramuscularly transplanted RLS_40_ cells were exposed to polychemotherapy in CHOP regimen on day 7 after transplantation (the 1st tumor passage). In 7 days after the 1st course of CHOP tumor cells were retransplanted into healthy animals and the 2nd course of CHOP was done (the 2nd tumor passage) (Figure 1, Scheme 1). In 10 days after the 2nd course of CHOP tumor cells were retransplanted into healthy animals and the 3rd course of CHOP was done (the 3rd tumor passage). Similarly the 4th tumor passage was carried out (Figure 1, Scheme 1). RLS_40_ undergone the same passages of retransplantation in the absence of CHOP was used as a control. This experimental model which is artificial as there is no counterpart in patients in clinical practice was developed to evaluate the impact of upregulated MDR of the tumor into hepatotoxicity in the absence of the damaging effect of multiple chemotherapies onto the liver. As the result of the treatment using Scheme 1 tumor was subjected to several courses of CHOP (from 1 to 4, in total 4), the liver was subjected to only one CHOP exposure.

During the treatment using Scheme 2 a group of mice with intramuscularly transplanted RLS_40_ cells was exposed to three subsequent courses of polychemotherapy in CHOP regimen (Figure 1, Scheme 2). On day 7 after tumor transplantation mice were exposed to CHOP (the 1st course); the 2nd and the 3rd courses were done with the interval of 7 days. Thus, both the tumor and liver have undergone triple exposure of chemotherapy. A group of mice with RLS_40_ without exposure to CHOP and a group of mice without tumor but exposed to CHOP were used as controls. This experimental model represents the real picture in clinical practice in patients undergoing multiple chemotherapies and allows us to study the effect of both multiple courses of polychemotherapy and deepening MDR of tumor onto hepatotoxicity.

### 3.3. Enhancement of MDR of RLS_40_ Cells due to Polychemotherapy: Alteration in Expression of the Genes Involved in the Development of MDR Phenotype

Levels of expression of the genes involved in the formation of multidrug resistance were estimated to confirm the enhancement of MDR of tumor after multiple chemotherapies. As expected after the treatment both at Scheme 1 and Scheme 2 the increase in expression of these genes was observed.

**Figure 2:**
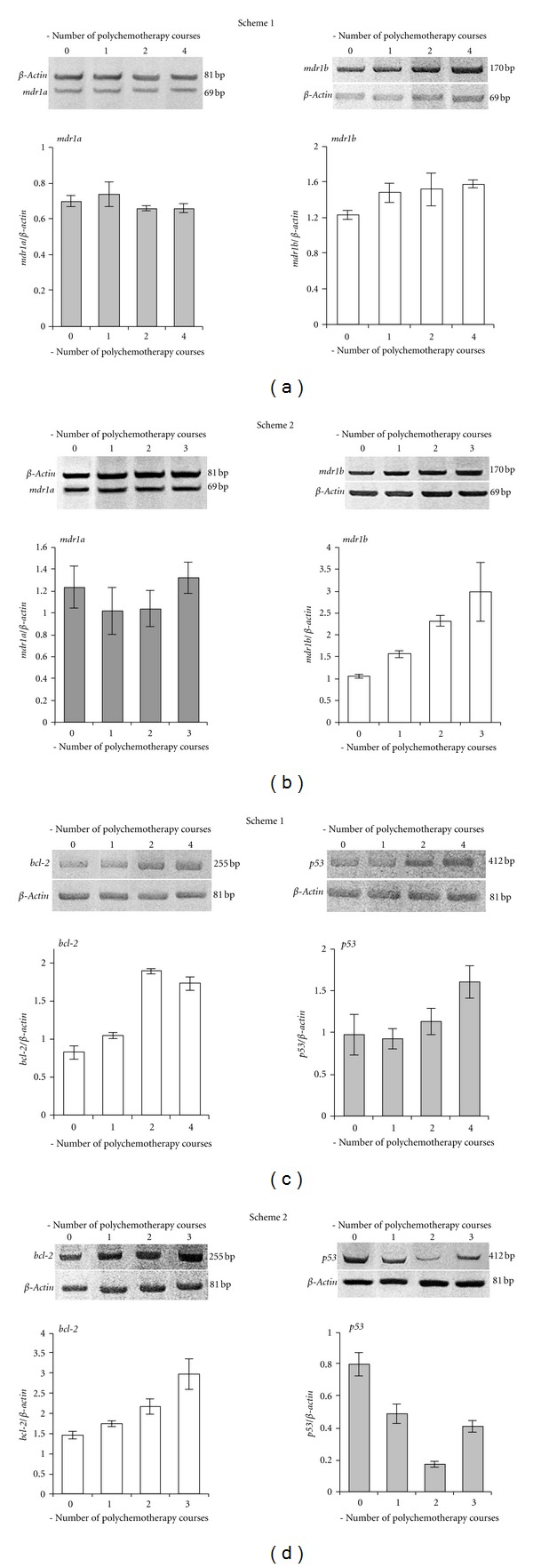
Expression levels of *mdr1a* (a, b), *mdr1b* (a, b), *bcl-2* (c, d), and *p53* (c, d) genes in cells of primary culture RLS_40_ after the treatment of tumor-bearing animals, according to Scheme 1 and Scheme 2. Separation of RT-PCR products in 8% PAAG and quantification of the band intensities are shown.

At baseline RLS_40_ cells were characterized by high expression levels of *mdr1a*, *mdr1b,* and *p53* genes and low expression level of *bcl-2* gene (Figure 2) that correlated with the data obtained earlier [13, 15]. The treatment under Scheme 1 resulted in 1.3- and 2.2-fold increases of the expression levels of *mdr1b* and *bcl-2* genes, respectively, (Figure 2(a), right panel, and Figure 2(c), left panel). The treatment under the Scheme 2 caused 2.8- and 2-fold increases of the expression of these genes, respectively, (Figure 2(b), right panel, and Figure 2(d), left panel).

However, the treatment under Scheme 1 and Scheme 2 differently affected the expression level of *p53* gene (Figures 2(c) and 2(d), right panels). After the treatment under Scheme 1 after two and four courses of polychemotherapy the expression levels of *p53* gene in RLS_40_ cells increased 1.2- and 1.6-fold, respectively, in comparison with the baseline. After the treatment under Scheme 2 after the 1st and the 2nd courses a 4.4-fold drop of the expression level of *p53* gene was seen in comparison with the baseline, but after the 3rd course gene expression it was slightly increased again.

Neither the treatment under Scheme 1 nor the treatment under Scheme 2 has an effect on the expression level of *mdr1a* gene (Figures 2(a) and 2(b), left panels), which was in consistent with earlier data that this gene poorly responded to the treatment [13, 15].

Alteration in the expression of *mdr1b*, *bcl-2,* and *p53* genes after the treatment that has been observed both at the artificial experimental conditions and at “real-life” situation of MDR status enhanced due to multiple chemotherapies in hematological patients which indicates not only the strengthening of MDR, but also abnormalities of the apoptosis mechanism [26–28].

**Figure 3:**
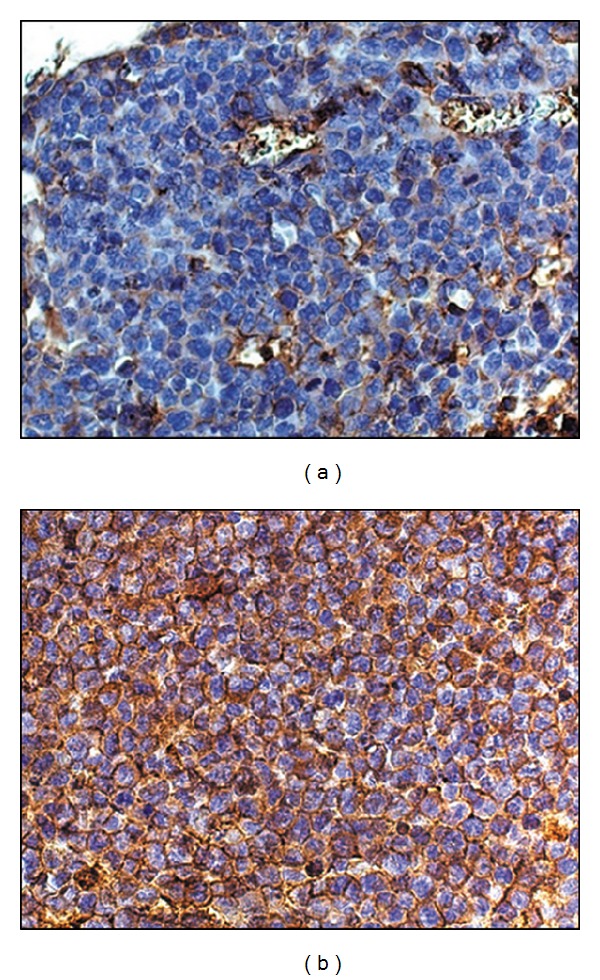
Pgp expression on the membrane of RLS_40_ cells at baseline (a) and after the 4th course of CHOP treatment according to Scheme 1 (b). Immunohistochemical staining of paraffin sections by Pgp mAb. Magnification ×100.

As the P-glycoprotein level on the surface membrane of tumor cells displays MDR of tumor [29–31] we estimated Pgp representation on the surface of the tumor before and after the treatment. It is seen that about 30% of RLS_40_ cells were initially Pgp positive (Pgp+) (Figure 3(a)). As expected multiple chemotherapies resulted in the significant increase in Pgp level on the membrane of tumor cells [32]. The treatment under Scheme 1 caused the significant increase in the number of Pgp+ cells in tumor: from 30% at baseline to 55.5 ± 1.5% after the 1st course and to 88.2 ± 1.5% after the 4th course of CHOP (Figure 3(b)). The treatment under Scheme 2 also resulted in the increase in Pgp level on the membrane of tumor cell: after the 1st and the 2nd courses of CHOP the number of Pgp+ cells was similar (60.9 ± 0.5% and 63.3 ± 0.5%, resp.), but after the 3rd the number of Pgp+ cells significantly increased up to 85.9 ± 1%.

### 3.4. The Effect of the Treatment under Schemes 1 and 2 on Tumor Growth of Mice with RLS_40_

**Figure 4:**
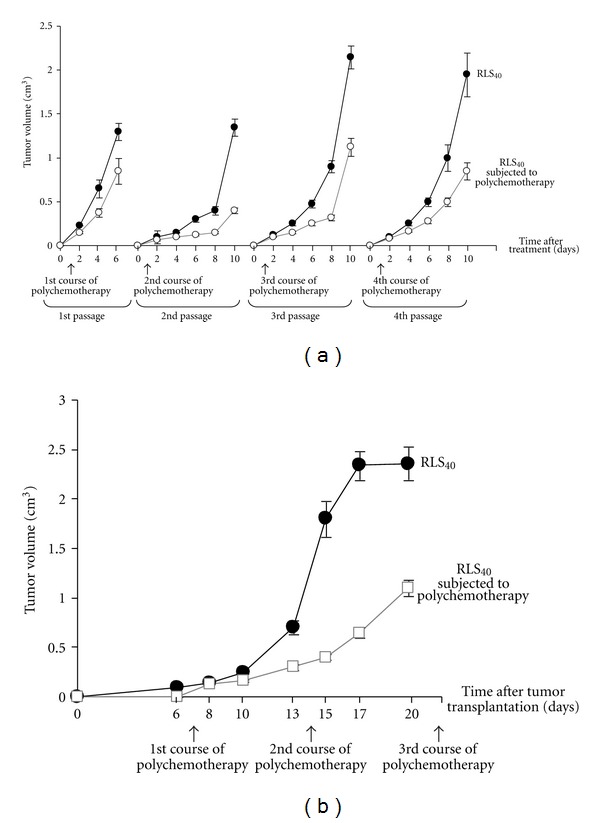
Dynamics of RLS_40_ growth in mice exposed to the treatment according to Scheme 1 (a) and Scheme 2 (b). Black circles indicate control, growth of RLS_40_ without treatment, brown squares indicate growth of RLS_40_ exposed to polychemotherapy, and arrows indicate the dates of polychemotherapy. On the scale of a day 1 after the treatment corresponds to day 8 after tumor implantation.

Dynamics of tumor growth in mice exposed to the CHOP treatment according to Scheme 1 and Scheme 2 were approximately the same regardless of the treatment regimen (Figure 4). Reliable inhibition of RLS_40_ tumor growth occurred upon the treatment both with Scheme 1 and Scheme 2, though RLS_40_ is not entirely regressed (Figures 4(a) and 4(b)). The inhibition of tumor growth depends on the number of treatment courses: after the 1st course of CHOP at Scheme 1 the inhibition of tumor growth was 36.6%, after the 2nd 58.7% (Figure 4(a)). The 3rd and the 4th courses of CHOP did not result in more pronounced inhibition of tumor growth: the inhibition rates were 50.5% and 45.5%, respectively, (Figure 4(a)). After the treatment under Scheme 2, reasonably similar effects were observed independent of the number of CHOP courses and the inhibition of tumor growth was approximately 50%. (Figure 4(b)).

### 3.5. Immunomodulatory Effect of CHOP Treatment

In the serum of mice bearing RLS_40_ without any treatment the increase in IFN-*γ* (up to 200 pg/mL) and IL-6 (up to 460 pg/mL) in comparison with healthy animals was observed. Two courses of CHOP treatment resulted in a strong activation of inflammatory response that should be considered as a negative event and an increase of interferons was regarded as a positive immunomodulatory activity. We observed statistically significant 8-fold increase in IL-1*α* (up to 240 pg/mL), 2-fold increase in IL-6 (up to 830 pg/mL), 20-fold increase in TNF-*α* (up to 110 pg/mL), 7.5-fold increase in INF-*γ* (up to 1500 pg/mL), and 9-fold increase in INF-*α* (up to 90 pg/mL).

### 3.6. The Effect of Polychemotherapy and Enhancement of MDR of Tumor onto the Liver

#### 3.6.1. Assessment of Destructive Changes and Regeneration Capacity of the Liver

Several factors can contribute to hepatotoxicity including endogenous intoxication caused by primary tumor node, metastatic infiltration of the liver, polychemotherapy itself and induced cytokine storm, and enhanced MDR of tumor. Since the basic idea of our work was the assumption that the upregulation of MDR of the tumor negatively affects the liver, we evaluated morphological changes and regeneration capacity of the liver depending on the number of the polychemotherapy courses affected the liver and the enhancement of MDR of the tumor.

Diffuse and focal infiltration of the liver by tumor cells was detected in tumor-bearing mice both without any treatment and subjected to the cytostatic treatment independently from the experimental scheme. First metastatic nodes appeared in the liver on day 10 after tumor transplantation. Volume density of metastasis in the liver on day 17 after tumor transplantation reached 21 ± 4% in the control group and 18 ± 3% after the treatment under Scheme 1 but this difference was statistically insignificant. In the case of Scheme 2 hepatic lymphoma infiltration progressively increased and reached up to 30 ± 5% of the entire liver parenchyma to day 24 after tumor transplantation both in groups after treatment and without it.

Upon the development of RLS_40_ without any treatment metastatic infiltration of the liver by tumor cells was accompanied with severe destructive changes increased with tumor progression: from 19 ± 0.9% (on day 8 after transplantation) to 54.2 ± 1.3% (on day 21 after transplantation). At the initial phase of tumor development alterative changes in the liver tissue were predominantly presented by degeneration of hepatocytes (11.4 ± 0.7% degeneration, 7.6 ± 0.7% necrosis). At a later date necrotic changes of the liver parenchyma were predominated (19.8 ± 1% degeneration, 34.4 ± 1.3% necrosis). The numerical density of binuclear hepatocyte decreased 1.5-fold (from 0.85 ± 0.15 to 0.55 ± 0.1), that shows a decline in the regeneration of liver tissue during tumor progression.

In the early stage of tumor progression (day 8 after transplantation) in the group of animals with RLS_40_ subjected to CHOP treatment a lesser pronounced infiltration of the liver parenchyma by tumor cells was identified, than in the control group (tumor-bearing animals without treatment). However, the volume density of destructive changes increased 1.5-fold in comparison with the control (28.8 ± 1% versus 19 ± 0.9% on day 8 after transplantation). At the latter stage of tumor progression (day 21 after tumor transplantation) in the same group of animals the volume density of alterative changes represented mainly by necrosis of the liver parenchyma continued to increase, but in a lesser extent in comparison with the control (tumor-bearing mice without any treatment; 51.2 ± 0.8% versus 54.2 ± 1.3%). The numerical density of binuclear hepatocytes changed insignificantly (from 0.7 ± 0.2 to 0.5 ± 0.1).

Comparison of the morphological changes developed in the liver to one and the same date at sequential passages of the tumor cells according to the Scheme 1 without treatment revealed a 1.8-fold increase in necrosis in the liver parenchyma after the 4th passage in comparison with the 1st passage of the tumor without treatment, while the level of degeneration in the liver parenchyma remained unchanged (Table 1). The numerical density of binuclear hepatocyte decreased 2-fold (from 1 ± 0.2 to 0.5 ± 0.1) after the 4th passage in comparison with the 1st passage.

Comparison of the morphological changes developed in the liver to one and the same day after tumor transplantation and CHOP treatment at sequential passages of the tumor cells according to the Scheme 1 revealed changes in the proportion of destructive changes in the liver of experimental animals from one passage to another. After the 1st passage (one course of CHOP for the tumor and one for the liver) the percentage of degenerative and necrotic cells was similar. While after the 4th passage (four courses of CHOP for the tumor and only one for the liver) the volume density of necrotic changes in the liver increased 1.3-fold and the volume density of degenerative change decreased 1.4-fold in comparison with the 1st passage of the tumor, which attests to the possible transformation of degeneration into necrosis (Table 1). The numerical density of binuclear hepatocytes decreased 1.8-fold after the 4th passage in comparison with the 1st one (from 0.9 ± 0.1 to 0.5 ± 0.1).

After three sequential courses of CHOP treatment (Scheme 2) both in animals with transplanted tumors and without tumors severe destructive changes in the liver parenchyma were revealed, but the dynamics of these changes in experimental groups were quite different.

On day 1 after the 1st course of CHOP in healthy mice the volume density of destructive changes in liver parenchyma consisting predominantly of degenerative hepatocytes was 50.8 ± 2.3%; on day 3 and day 7 these values decreased 1.3- and 1.8-fold, respectively, (Table 2). On day 1 and day 3 both after the 2nd and the 3rd courses of CHOP the percentage of destructive changes in liver parenchyma did not differ significantly from the value after the 1st course of CHOP; however, on day 7 both after the 2nd and the 3rd courses of CHOP these values increased 1.7- and 1.8-fold, respectively, as compared to the 1st course of CHOP administered to healthy mice (Table 2).

On day 1 and day 3 after the 1st course of CHOP under Scheme 2 in mice with RLS_40_ volume densities of destructive changes in liver parenchyma were 53.8 ± 3% and 39.8 ± 2%, respectively. These changes were presented predominantly by degenerative hepatocytes and did not differ significantly from healthy mice exposed to chemotherapy at the same regimen. On day 7 after the 1st course of CHOP in tumor-bearing mice the percentage of destructive changes in liver parenchyma increased 1.6-fold in comparison with the healthy mice exposed to the same treatment. After remaining courses of CHOP destructive changes in liver tissue of tumor-bearing mice continued to increase progressively and as a result amounted to 85% of liver parenchyma (Table 2).

**Figure 5:**
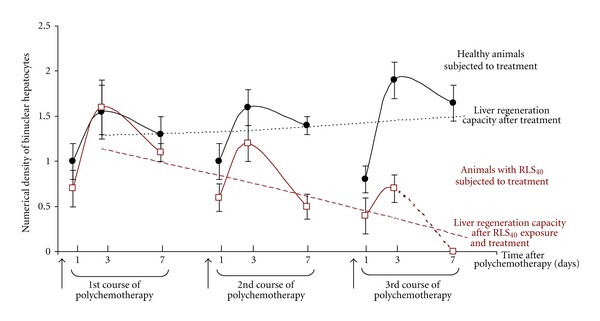
Kinetics of the numerical density of binuclear hepatocytes in the liver tissue of healthy mice or mice bearing RLS_40_ after polychemotherapy. The treatment of healthy mice and mice with RLS_40_ was performed according to Scheme 2. The resulting lines displaying the liver regeneration capacity were formed using the average values of numerical density of binuclear hepatocytes for each course of polychemotherapy.

To evaluate the toxic effect of multiple chemotherapies and tumor with advanced MDR on the regeneration capacity of the liver the numerical density of binuclear hepatocytes was measured in the groups of experimental animals subjected to the treatment under Scheme 2 with or without tumor (Figure 5).

Maximal number of binuclear hepatocytes is observed on 1-2 days after any damage (alcohol, chemotherapy, CCl_4_, LPS). Hepatocyte degeneration can turn into either necrosis or regenerate and become normal hepatocytes due to the potential of binuclear hepatocytes. For all groups of animals the bell-shaped kinetics of numerical density of binuclear hepatocytes was observed: no effect on day 1, the maximal regeneration burst was observed on day 3, and decrease in numerical density was observed on day 7 after polychemotherapy. In the group of healthy animals subjected to polychemotherapy the regeneration capacity did not depend on the number of CHOP courses (1.55 ± 0.3, 1.6 ± 0.2, and 1.9 ± 0.2 at the day 3 after the 1st, the 2nd, and the 3rd courses of CHOP, resp.). However, in the group of animals bearing RLS_40_ each subsequent course of CHOP resulted in the progressive decrease in regeneration capacity of the liver: the numerical density of binuclear hepatocytes was 1.6 ± 0.3, 1.2 ± 0.2, and 0.7 ± 0.15 at the day 3 after the 1st, the 2nd, and the 3rd courses of chemotherapy (Figure 5). Volume density of metastasis in the liver reached 30% at the end of the experiment and also contributed to the drop of regeneration capacity of the liver.

During the RLS_40_ passaging without treatment (Scheme 1, control group) the numerical density of binuclear hepatocytes did not change between the passages and was 1 ± 0.15 (day 14 after tumor transplantation). During the RLS_40_ passaging with polychemotherapy (Scheme 1, experimental group) the numerical density of binuclear hepatocytes also did not change between the passages and was 0.8 ± 0.1 (day 14 after tumor transplantation, day 7 after treatment).

The obtained data point to combined (synergic) toxic effects of the endogenous intoxication caused by primary tumor node, metastatic infiltration of the liver, multiple chemotherapies, and upregulated MDR of the tumor onto the liver that results in the significant destructive changes in the liver tissue and substantial reduction of the regenerative capacity of the liver.

#### 3.6.2. P-Glycoprotein Expression in the Liver Tissue

It was shown that the liver tissue of healthy mice without any treatment is characterized by Pgp expression [33]: the percentage of hepatocytes with Pgp 2+ and Pgp 3+ was about 40% of the entire population of liver cells. After three subsequent courses of CHOP (Scheme 2) in healthy mice the increase in Pgp expression on hepatocyte membrane was detected: after the 1st course of CHOP the total percentage of hepatocytes with Pgp 2+ and Pgp 3+ was 55.8 ± 0.5%, after the 2nd course 58.3 ± 0.5%, and after the 3rd course 63 ± 0.7%. Therefore, several courses of chemotherapy in healthy mice resulted in a gradual and slow increase in Pgp expression in the liver tissue. In contrast to healthy mice three courses of CHOP in tumor-bearing mice (Scheme 2) caused a rapid and more pronounced increase in Pgp expression: after each course of CHOP the percentage of hepatocytes with Pgp 2+ and Pgp 3+ was about 70% of the entire population of liver cells.

**Figure 6:**
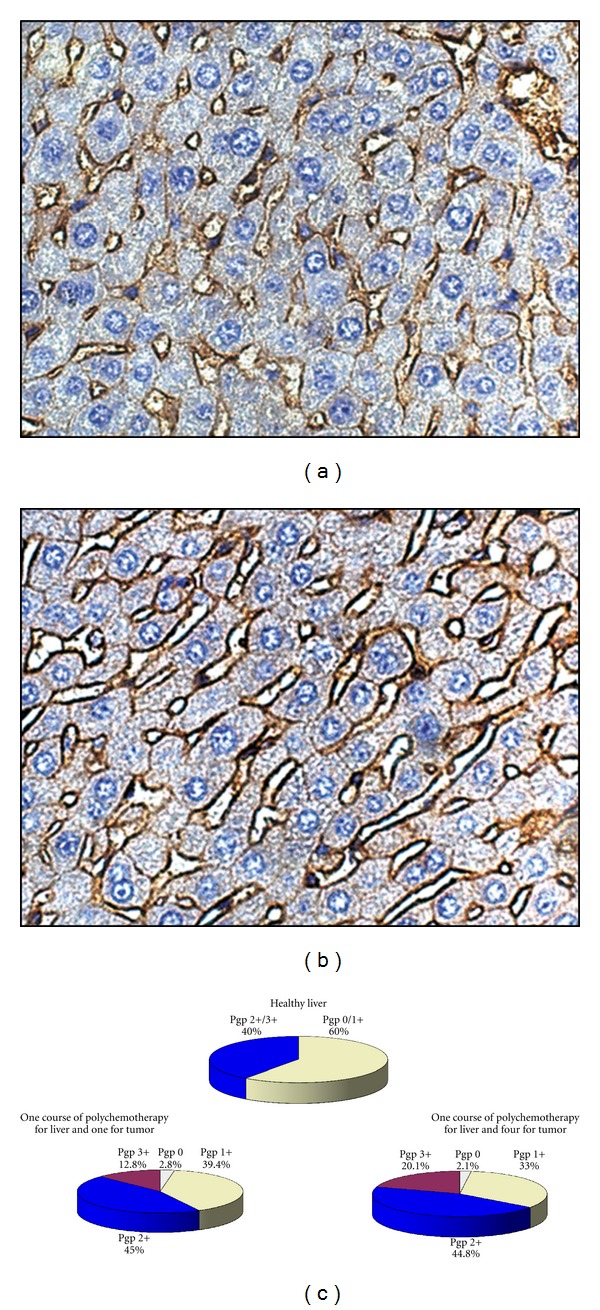
P-Glycoprotein expression on the surface of hepatocyte membrane in the liver. Immunohistochemical staining of paraffin sections of the liver of healthy mice (a) and mice bearing RLS_40_ after multiple polychemotherapy according to Scheme 1 (b). Quantification of the results of immunohistochemical staining (c). Expression levels correspond to the following scale: 0 no expression, 1+ weak expression, 2+ moderate expression, and 3+ strong expression.

After the treatment of tumor-bearing animals under Scheme 1 (four courses of CHOP for the tumor and only one for the liver) the increase in hepatocyte percentage with Pgp 2+ and Pgp 3+ was also detected. After the 1st tumor passage with the following cytostatic treatment percentage of hepatocyte with Pgp 2+ and Pgp 3+ was 57.8 ± 0.45%, after the 2nd 59.8 ± 0.5%, after the 3rd 63.3 ± 0.5%, and after the 4th 64.9 ± 0.3% (Figure 6). Therefore, despite the fact that the liver is exposed to only one course of chemotherapy, the upregulation of MDR of the tumor makes a contribution to the increase in Pgp expression in the liver tissue after cytostatic influence.

## 4. Discussion

Progress in antitumor therapy of hematological malignances achieved over the past decades resulted in an increase in the amount of complete remission, progression-free survival, and recovery in some cases. These results are due to the use of effective chemotherapy protocols based on the decrease in intervals between chemotherapy courses, increase in cytostatic dose, and introduction of additional chemotherapeutic agents into the protocol. However, the high toxicity of multiple courses of chemotherapy and multidrug resistance of tumor cells usually typical for hematological malignancies even at the baseline are important obstacles significantly reducing the efficiency of the treatment.

The potential of antitumor chemotherapy is often limited by hepatotoxicity of cytotoxic drugs undergoing biotransformation in the liver [11, 12]. The risk of toxic complications due to high concentrations of cytostatic metabolites is increased by tumor intoxication and metastatic lesion of the liver [34]. Toxic damage of the liver impedes the long-term treatment, reduces the efficiency of cytostatic therapy, and requires constant correction by the hepatotropic drugs in the intervals between the treatment courses [35].

A number of factors may contribute to the toxic effects of chemotherapy (genetically predisposed individual features of drug metabolism, the presence of comorbidity, and other factors affecting the detoxication function of the liver) [36, 37]. P-Glycoprotein-mediated multidrug resistance of tumor may be one of these factors due to the increasing burden of cytostatics onto the liver through the drug pumping out of tumor cells.

Herein, we studied the combined effect of polychemotherapy (CHOP protocol) and lymphosarcoma RLS_40_ characterized by enhanced MDR onto the liver of experimental animals using two treatment schemes. Scheme 1 is an artificial and has no analogues in clinical practice. According to Scheme 1 four courses of CHOP for tumor were completed, but only one was applied for the liver. Scheme 2 reflects a real-life situation, where tumor-bearing mice were exposed to three subsequent courses of CHOP, so both the liver and the tumor have three CHOP courses.

In the case of RLS_40_ tumor model several factors can contribute to hepatotoxicity: endogenous intoxication caused by primary tumor node, metastatic infiltration of the liver, polychemotherapy itself and induced cytokine storm, and enhanced MDR of the tumor. We attempted to evaluate the contribution of the multiple chemotherapies and the strengthened MDR of the tumor to hepatotoxicity as well as the combined effect of the multiple chemotherapies and the tumor with upregulated MDR on the liver.

As it was expected the enhancement of MDR of the tumor at the treatment under Scheme 1 and Scheme 2 was observed. MDR of RLS_40_ at baseline is provided by high levels of Pgp and Bcl-2 [12]. Multiple chemotherapies cause an additional increase in *mdr1b* and *bcl-2* gene expression in RLS_40_ cells that makes the tumor less sensitive to cytostatic treatment [27, 38]. We also confirmed that multiple chemotherapies in different regimens caused the significant increase in Pgp expressions on the membrane of tumor cells up to 90% of the entire tumor tissue.

We evaluated the destructive changes and regeneration capacity of the liver that reflect the degree of hepatic dysfunction. Morphological analysis of the liver of tumor-bearing mice both without treatment and after CHOP treatment revealed its diffuse and focal infiltration by tumor cells, which by itself is a liver damaging factor.

At the time of tumor development without any treatment infiltration of liver tissue by tumor cells was accompanied by severe destructive changes due to both the tumor invasion and to mediated influence of the growing tumor on the host by endogenous intoxication [39]. The livers of mice with RLS_40_ subjected to CHOP are characterized by an increase in the total destructive changes, occupying more than 80% of liver parenchyma, in comparison with healthy animals without tumors subjected to CHOP treatment and tumor-bearing animals without any treatment. Differences in the destructive changes in the liver confirm that multiple chemotherapies and tumor with upregulated MDR have a synergistic damaging effect on the liver.

One of the most important characteristics of the liver is its regeneration capacity, the morphological expression of which is the numerical density of binuclear hepatocytes. Chemotherapy in the absence of tumor virtually does not affect the numerical density of binuclear hepatocytes after triple exposure (Scheme 2), which points to the maintenance of regeneration capacity of the liver. But the strengthening of MDR of RLS_40_ after each subsequent course of chemotherapy (Scheme 2) together with metastasis progression reduces the numerical density of binuclear hepatocytes and impairs the liver regeneration capacity. Treatment under Scheme 1 also leads to the decrease in the regeneration capacity of the liver, but less significantly.

Together with the pressure of multiple CHOP, hepatic lymphoma infiltration and the enhancement of MDR of the tumor one of the reasons for decline of the regeneration capacity of the liver may be the synergic rising of toxic burden. It takes place because of the strengthening of multidrug resistance of the tumor that considering the large tumor mass relative to body weight leads to increased concentrations of cytostatics due to their force pumping out of tumor cells. Components of CHOP treatment are released into the bloodstream and as a result the liver does not handle the metabolic burden. Although the level of Pgp in the liver increases it is not sufficient to protect hepatocytes which is confirmed by an increase in destructive changes. Additionally the increase in necrotizing sites within the tumor also contributes into the toxic burden on the liver. Therefore, hepatotoxic effects may become stronger with the progression of MDR of the tumor.

The liver tissue itself has rich system of membrane transporters, proteins involved in the absorption, metabolism, distribution, and excretion of xenobiotics [40, 41], providing the maintenance and restoration of its own liver structure and this function is partly mediated by Pgp [42–44]. A number of chemicals, including cytotoxic drugs used in multiple dosing regimens, may cause an alteration in P-glycoprotein expression in different tissues (liver, kidney, and intestine) and lead to a change in drug pharmacokinetics [45–47].

In our study we showed that exposure of healthy animals to CHOP in full regimen (under Scheme 2, three courses) caused a slow and gradual increase in P-glycoprotein expression on the membrane of hepatocytes. At the same time exposure of animals with RLS_40_ to CHOP under Scheme 2 irrespective of the course number resulted in a rapid and more pronounced increase in Pgp expression. Our data also revealed a small but reliable increase in Pgp expression in the liver tissue of animals having one CHOP course onto the liver and four CHOP courses onto the tumor (Scheme 1) in comparison with animals having one CHOP course onto the liver and one CHOP course onto the tumor. Such alteration in the expression of transporter proteins may represent a compensatory mechanism after the toxic effect of the chemotherapy onto the liver enhanced by the upregulated MDR of the tumor [48]. The increase of the concentration of transporters in the membrane of hepatocytes may reduce the accumulation of potentially toxic chemicals [49–51]. But in the used experimental models it was not sufficient to protect hepatocytes from further damage, what we observed as an increase in destructive changes.

Our data suggest that MDR status of a tumor may be one of the damaging factors, which contributes to toxic liver burden during polychemotherapy.

## 5. Conclusion

Progression of multidrug resistance of the tumor negatively affected the liver of the host and caused strong hepatotoxic effects manifested by the accumulation of degenerative changes of hepatocytes and the reduction in regeneration capacity of the liver. These data suggest that MDR status of the tumor at baseline may contribute to toxic liver burden and serve as a negative predictor parameter of hepatic dysfunction. The toxic effects of multiple chemotherapies onto the liver are accelerated by strengthening MDR of the tumor that should be taken into account when developing therapeutic regimens of malignancies with upregulated MDR.

In this context, it is relevant to develop approaches for overcoming or reducing unfavorable toxic effects caused by polychemotherapy in combination with the upregulated MDR of the tumor. This can be achieved through the development of individual treatment protocols with detection of MDR status of the tumor prior to therapy and overcoming MDR using modern molecular gene-targeted approaches.

## Figures and Tables

**Table 1:** Morphometric analysis of liver tissue of mice with RLS_40_ subjected to the treatment under Scheme  1.

Passage number	Hepatocyte degeneration, Vv, % (Mean ± SEM)			Hepatocyte necrosis, Vv, % (Mean ± SEM)		
	Healthy subjected to CHOP (*n* = 10)	RLS_40_ without treatment (*n* = 15)	RLS_40_ subjected to CHOP (*n* = 15)	Healthy subjected to CHOP (*n* = 10)	RLS_40_ without treatment (*n* = 15)	RLS_40_ subjected to CHOP (*n* = 15)
Baseline*	7.3 ± 0.7	11.4 ± 0.7		3.1 ± 0.5	7.6 ± 0.7	
1^†^	15.7 ± 0.9	16.1 ± 0.8	21.9 ± 0.7^#^	12.6 ± 1	19.1 ± 1.0	23.1 ± 0.8^#^
2^‡^	17.1 ± 1.0	22.2 ± 1.2^∧^	18.8 ± 0.7^#∧^	10.0 ± 0.8	25.1 ± 0.9^∧^	25.9 ± 0.9
3^‡^	15.4 ± 1.0	16.2 ± 0.6	15.1 ± 0.7^∧^	12.0 ± 0.8	29.7 ± 1.0^∧^	27.7 ± 0.9^∧^
4^‡^	16.0 ± 1.2	17 ± 0.8	15.5 ± 0.9^∧^	13.2 ± 1.2	33.8 ± 1.3^∧^	30.6 ± 1.0^#∧^

*Initial level determined just before treatment (6th day after tumor transplantation).

^†^At day 7 after the treatment.

^‡^At day 10 after the treatment.

^∧^ *P* ≤ 0.05 compared to the 1st passage within one group.

^#^ *P* ≤ 0.05 compared to the group without treatment.

**Table 2:** Morphometric analysis of liver tissue in healthy mice and mice with RLS_40_ after the treatment under Scheme  2.

PCHT courses, number	Days after treatment	Hepatocyte degeneration, Vv, % (Mean ± SEM)		Hepatocyte necrosis, Vv, % (Mean ± SEM)		Volume density of hepatic lymphoma infiltration, Vv, %
		Healthy mice	Mice with RLS_40_	Healthy mice	Mice with RLS_40_	
		(*n* = 45)	(*n* = 45)	(*n* = 45)	(*n* = 45)	
Baseline^#^		7.3 ± 0.7	12.6 ± 1*	3.1 ± 0.5	12.1 ± 1*	—

1	1	33.7 ± 2.2	40.7 ± 3.2	17.1 ± 1.1	13.5 ± 1.2*	—
	3	24 ± 2.1	20.6 ± 1.6	16.4 ± 1.4	19.2 ± 1.3	—
	7	15.7 ± 0.9	27.7 ± 1.6*	12.6 ± 1	18 ± 1.35*	15 ± 4

2	1	27.5 ± 1.5	25.9 ± 1.2	19.7 ± 1.7	24 ± 1*	—
	3	26.6 ± 1.7	40.7 ± 2.1*	17.6 ± 1.4	20.8 ± 1.3	—
	7	31.4 ± 1.8	29.6 ± 1.9	17 ± 1	37.9 ± 3.7*	26 ± 4

3	1	27.2 ± 1.2	34.3 ± 3*	13.6 ± 1.3	40.8 ± 3.6*	—
	3	27.9 ± 1.3	36.9 ± 2.6*	9.6 ± 0.9	47.7 ± 4.1*	30 ± 5
	7	28.7 ± 1.2	—	22.9 ± 1.2	—	—

*P ≤ 0.05 compared to healthy mice.

^#^Initial level determined just before treatment (6th day after tumor transplantation).

## References

[1] Sanderson H, Brain RA, Johnson DJ, Wilson CJ, Solomon KR (2004). Toxicity classification and evaluation of four pharmaceuticals classes: antibiotics, antineoplastics, cardiovascular, and sex hormones. *Toxicology*.

[2] Wollmer E, Neubauer A (2011). Side effects of tumor pharmacotherapy. What internists should know. *Internist*.

[3] Marques DS, Sandrini JZ, Boyle RT, Marins LF, Trindade GS (2010). Relationships between multidrug resistance (MDR) and stem cell markers in human chronic myeloid leukemia cell lines. *Leukemia Research*.

[4] Tsiftsoglou AS, Pappas IS, Vizirianakis IS (2003). Mechanisms involved in the induced differentiation of leukemia cells. *Pharmacology and Therapeutics*.

[5] Shtil AA (2002). Emergence of multidrug resistance in leukemia cells during chemotherapy: mechanisms and prevention. *Journal of Hematotherapy and Stem Cell Research*.

[6] Stavrovskaya AA (2000). Cellular mechanisms of multidrug resistance of tumor cells. *Biochemistry*.

[7] Stavrovskaya AA, Stromskaya TP (2008). Transport proteins of the ABC family and multidrug resistance of tumor cells. *Biochemistry*.

[8] Maung ZT, MacLean FR, Reid MM (1994). The relationship between bcl-2 expression and response to chemotherapy in acute leukaemia. *British Journal of Haematology*.

[9] Filipits M (2004). Mechanisms of cancer: multidrug resistance. *Drug Discovery Today*.

[10] Chauhan PS, Bhushan B, Singh LC (2011). Expression of genes related to multiple drug resistance and apoptosis in acute leukemia: response to induction chemotherapy. *Experimental and Molecular Pathology*.

[11] King PD, Perry MC (2001). Hepatotoxicity of chemotherapy. *Oncologist*.

[12] Jaeschke H, Gores GJ, Cederbaum AI, Hinson JA, Pessayre D, Lemasters JJ (2002). Mechanisms of hepatotoxicity. *Toxicological Sciences*.

[13] Mironova N, Shklyaeva O, Andreeva E (2006). Animal model of drug-resistant tumor progression. *Annals of the New York Academy of Sciences*.

[14] Chattopadhyay N, Kher R, Godbole M (1993). Inexpensive SDS/phenol method for RNA extraction from tissues.. *BioTechniques*.

[15] Patutina OA, Mironova NL, Popova NA (2010). The siRNA targeted to mdr1b and mdr1a mRNAs in vivo sensitizes murine lymphosarcoma to chemotherapy. *BMC Cancer*.

[16] Viktorov IV, Proshin SS (2003). Use of isopropyl alcohol in histological assays: dehydration of tissue, enbessing into paraffin, and processing of paraffin sections. *Bulletin of Experimental Biology and Medicine*.

[17] Buesa RJ, Peshkov MV (2009). Histology without xylene. *Annals of Diagnostic Pathology*.

[18] Weibel ER (1979). *Stereological Methods. Practical Methods for Biological Morphometry*.

[19] Avtandilov GG (1990). *Medical Morphometry*.

[20] True LD (1996). Morphometric applications in anatomic pathology. *Human Pathology*.

[21] Pascahle CL, Miller MC, Chiu C (2011). Amyloid-beta transporter expression at the blood-CSF barrier is age-dependent. *Fluids and Barriers of the CNS*.

[22] Hoffmann K, Löscher W (2007). Upregulation of brain expression of P-glycoprotein in MRP2-deficient TR-rats resembles seizure-induced up-regulation of this drug efflux transporter in normal rats. *Epilepsia*.

[23] Shi H, Lu D, Shu Y, Shi W, Lu S, Wang K (2008). Expression of multidrug resistance-related proteins p-glycoproteinglutathione-s-transferases, topoisomerase-II and lung resistance protein in primary gastric cardiac adenocarcinoma. *Cancer Investigation*.

[24] Petterino C, Rossetti E, Bertoncello D (2006). Immunohistochemical detection of P-glycoprotein (Clone C494) in canine mammary gland tumours. *Journal of Veterinary Medicine A*.

[25] Ng IOL, Liu CL, Fan ST, Ng M (2000). Expression of P-glycoprotein in hepatocellular carcinoma: a determinant of chemotherapy response. *American Journal of Clinical Pathology*.

[26] Zenkov AN, Scvortsova NV, Chernolovskaya EL, Pospelova TI, Vlassov VV (2004). Expression of the MDR1 and MRP genes in patients with lymphoma with primary bone marrow involvement. *Nucleosides, Nucleotides and Nucleic Acids*.

[27] Kasimir-Bauer S, Beelen D, Flasshove M, Noppeney R, Seeber S, Scheulen ME (2002). Impact of the expression of P glycoprotein, the multidrug resistance-related protein, bcl-2, mutant p53, and heat shock protein 27 on response to induction therapy and long-term survival in patients with de novo acute myeloid leukemia. *Experimental Hematology*.

[28] Gibalová L, Sereš M, Rusnák A (2012). P-glycoprotein depresses cisplatin sensitivity in L1210 cells by inhibiting cisplatin-induced caspase-3 activation. *Toxicol in Vitro*.

[29] Stouch TR, Gudmundsson O (2002). Progress in understanding the structure-activity relationships of P-glycoprotein. *Advanced Drug Delivery Reviews*.

[30] Kourti M, Vavatsi N, Gombakis N (2007). Expression of multidrug resistance 1 (MDR1), multidrug resistance-related protein 1 (MRP1), lung resistance protein (LRP), and breast cancer resistance protein (BCRP) genes and clinical outcome in childhood acute lymphoblastic leukemia. *International Journal of Hematology*.

[31] Komdeur R, Plaat BEC, Van Der Graaf WTA (2003). Expression of multidrug resistance proteins, P-gp, MRP1 and LRP, in soft tissue sarcomas analysed according to their histological type and grade. *European Journal of Cancer*.

[32] Oue T, Yoneda A, Uehara S, Yamanaka H, Fukuzawa M (2009). Increased expression of multidrug resistance-associated genes after chemotherapy in pediatric solid malignancies. *Journal of Pediatric Surgery*.

[33] Mahmood B, Daood MJ, Hart C, Hansen TWR, Watchko JF (2001). Ontogeny of P-glycoprotein in mouse intestine, liver, and kidney. *Journal of Investigative Medicine*.

[34] Ludwig R, Weirich A, Abel U, Hofmann W, Graf N, Tournade MF (1999). Hepatotoxicity in patients treated according to the nephroblastoma trial and study SIOP-9/GPOH. *Medical and Pediatric Oncology*.

[35] Floyd J, Mirza I, Sachs B, Perry MC (2006). Hepatotoxicity of chemotherapy. *Seminars in Oncology*.

[36] Gonzalez-Angulo AM, Morales-Vasquez F, Hortobagyi GN (2007). Overview of resistance to systemic therapy in patients with breast cancer. *Advances in Experimental Medicine and Biology*.

[37] Hartmann G, Kim H, Piquette-Miller M (2001). Regulation of the hepatic multidrug resistance gene expression by endotoxin and inflammatory cytokines in mice. *International Immunopharmacology*.

[38] Staibano S, Franco R, Tranfa F (2004). Orbital rhabdomyosarcoma: relationship between DNA ploidy, p53, bcl-2, MDR-1 and Ki67 (MIB1) expression and clinical behavior. *Anticancer Research*.

[39] Tretiak NM, Anoshina MI, Mnishenko VM (2001). Endogenous intoxication, immunological reactivity and erythrocyte membrane permeability in patients with chronic myelogenous leukemia. *Likars"ka sprava*.

[40] Mizuno N, Niwa T, Yotsumoto Y, Sugiyama Y (2003). Impact of drug transporter studies on drug discovery and development. *Pharmacological Reviews*.

[41] Endres CJ, Hsiao P, Chung FS, Unadkat JD (2006). The role of transporters in drug interactions. *European Journal of Pharmaceutical Sciences*.

[42] Tygstrup N, Bangert K, Ott P, Bisgaard HC (2002). Messenger RNA profiles in liver injury and stress: a comparison of lethal and nonlethal rat models. *Biochemical and Biophysical Research Communications*.

[43] Staud F, Ceckova M, Micuda S, Pavek P (2010). Expression and function of p-glycoprotein in normal tissues: effect on pharmacokinetics.. *Methods in Molecular Biology*.

[44] Sarkadi B, Homolya L, Szakacs G, Varadi A (2006). Human multidrug resistance ABCB and ABCG transporters: participation in a chemoimmunity defense system. *Physiological Reviews*.

[45] Gustafson DL, Long ME (2001). Alterations in P-glycoprotein expression in mouse tissues by doxorubicin: implications for pharmacokinetics in multiple dosing regimens. *Chemico-Biological Interactions*.

[46] Islam MO, Hara M, Miyake J (2002). Induction of P-glycoprotein, glutathione-S-transferase and cytochrome P450 in rat liver by atrazine. *Environmental Toxicology and Pharmacology*.

[47] Hartmann G, Vassileva V, Piquette-Miller M (2005). Impact of endotoxin-induced changes in P-glycoprotein expression on disposition of doxorubicin in mice. *Drug Metabolism and Disposition*.

[48] Kageyama M, Fukushima K, Togawa T (2006). Relationship between excretion clearance of rhodamine 123 and P-glycoprotein (Pgp) expression induced by representative Pgp inducers. *Biological and Pharmaceutical Bulletin*.

[49] Aleksunes LM, Scheffer GL, Jakowski AB, Pruimboom-Brees IM, Manautou JE (2006). Coordinated expression of multidrug resistance-associated proteins (Mrps) in mouse liver during toxicant-induced injury. *Toxicological Sciences*.

[50] Barnes SN, Aleksunes LM, Augustine L (2007). Induction of hepatobiliary efflux transporters in acetaminophen-induced acute liver failure cases. *Drug Metabolism and Disposition*.

[51] Fromm MF (2000). P-glycoprotein: a defense mechanism limiting oral bioavailability and CNS accumulation of drugs. *International Journal of Clinical Pharmacology and Therapeutics*.

